# Correction to “**Allergy to *Theobroma Cacao***”

**DOI:** 10.1002/ccr3.9201

**Published:** 2024-07-19

Nin Valencia A, Retes LS, Bartolomé‐Zavala B, Perdomo Gutiérrez G, Bigorra T. Allergy to Theobroma Cacao. Clin Case Rep. 2024 Jun 14;12(6):e8803.

In the article entitled “Allergy to Theobroma Cacao” that was previously published in Volume 12 Issue 6 of Clinical Case Reports, an author's name was incorrectly formatted in the citation.

Lorena Soto Rates' name should be formatted as **Soto Retes L.**

We apologize for this error.

